# *In silico* prediction and screening of modular crystal structures via a high-throughput genomic approach

**DOI:** 10.1038/ncomms9328

**Published:** 2015-09-23

**Authors:** Yi Li, Xu Li, Jiancong Liu, Fangzheng Duan, Jihong Yu

**Affiliations:** 1 State Key Laboratory of Inorganic Synthesis and Preparative Chemistry, Jilin University, Qianjin Street 2699, Changchun 130012, China

## Abstract

High-throughput computational methods capable of predicting, evaluating and identifying promising synthetic candidates with desired properties are highly appealing to today's scientists. Despite some successes, *in silico* design of crystalline materials with complex three-dimensionally extended structures remains challenging. Here we demonstrate the application of a new genomic approach to ABC-6 zeolites, a family of industrially important catalysts whose structures are built from the stacking of modular six-ring layers. The sequences of layer stacking, which we deem the genes of this family, determine the structures and the properties of ABC-6 zeolites. By enumerating these gene-like stacking sequences, we have identified 1,127 most realizable new ABC-6 structures out of 78 groups of 84,292 theoretical ones, and experimentally realized 2 of them. Our genomic approach can extract crucial structural information directly from these gene-like stacking sequences, enabling high-throughput identification of synthetic targets with desired properties among a large number of candidate structures.

## Abstract (web summary)

High-throughput computation aids design of new functional materials. Here, Yu *et al.* develop a high-throughput screening method for a group of zeolites with crystalline modular structures which are viewed as having gene-like stacking codes, and identify the most promising structures with desired properties.

## Graphical abstract

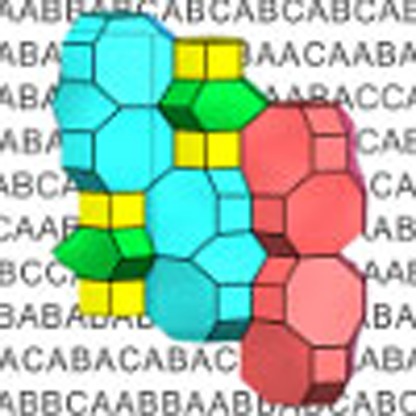

Discovering new advanced materials, which is one of the most important tasks for materials scientists and chemists, still relies primarily on scientific intuition and trial-and-error experimentation^1^. In 2011, the US White House launched the Materials Genome Initiative aiming to develop high-throughput computer methods and data-sharing systems to complement and fully leverage existing experimental research on advanced materials. The incorporation of new computer and informatics tools has the potential to accelerate materials innovation in: (1) predicting a large number of unknown candidate compounds^2^,^3^,^4^,^5^,^6^,^7^,^8^,^9^,^10^,^11^,^12^,^13^,^14^,^15^; (2) evaluating the predicted compounds and removing the unrealizable ones^16^,^17^,^18^,^19^,^20^,^21^; and (3) screening the predicted compounds and identifying synthetic candidates with desired properties^22^,^23^,^24^,^25^,^26^,^27^,^28^,^29^,^30^,^31^. Despite all these successes, *in silico* materials innovation is still facing many challenges. Unlike the genes of organisms, encoding and decoding the structural information of many important crystalline materials remains very complicated. Meanwhile, the explicit structure-property relationships for many materials are not yet clear, so high-throughput identification of synthetic targets with desired properties among a large number of candidate structures is still challenging.

Fortunately, the structures of many crystalline materials can topologically be decomposed into a set of smaller and simpler building modules. In particular, many materials are built of well-defined parallel-stacked modular layers^14^,^32^,^33^. If each unique layer is assigned a predefined symbol, then the stacking of these layers can be expressed as a sequence of predefined symbols, just like the genes of organisms. Since each stacking sequence uniquely identifies a specific three-dimensional structure, we deem it the gene of the corresponding structure. Such gene-like one-dimensional stacking sequences can be easily processed by computers, so high-throughput enumeration, evaluation, and identification of theoretical structures with desired properties will be accessible. In this contribution, for the first time, we demonstrate the application of a new genomic approach to ABC-6 zeolites, a family of industrially important catalysts constructed from the stacking of modular 6-ring layers.

To date, over 150 types of ABC-6 zeolites with 28 distinct framework topologies have been discovered, among which cancrinite, sodalite and chabazite are the best-known representatives (Supplementary Table 1). The frameworks of all ABC-6 zeolites can be decomposed into parallel six-ring layers stacked along the *c*-direction in hexagonal unit cells, and the vertices of each 6-ring are corner-sharing TO_4_ tetrahedra (T=Si, Al, or P and so on). An ABC-6 structure may consist of three types of six-ring layers, which are centred at the (0,0,*z*), (1/3,2/3,*z*), and (2/3,1/3,*z*) axes, respectively. If we denote these three types of layers by letters A, B, and C, then the stacking sequences for cancrinite, sodalite and chabazite will be (AB), (ABC), and (AABBCC), respectively (Fig. 1). Meanwhile, the stacking of six-rings gives rise to various types of well-defined polyhedral cages in molecular dimensions, which are the most important structural features for ABC-6 zeolites (to avoid confusion, the stacking sequences for these polyhedral cages are given in lower case throughout this paper). These featured cages may hold various types of extraframework cations, anion groups and/or water molecules, which can be exchanged or removed, providing void space suitable for the adsorption, diffusion and reaction of many types of guest species^34^,^35^,^36^,^37^,^38^,^39^,^40^. For instance, chabazite and its synthetic counterparts are able to trap CO_2_ in their featured cages, showing the highly desired capability for carbon capture from the atmosphere^36^,^37^; meanwhile, these zeolites are currently among the best industrial catalysts for methanol-to-olefin (MTO) reactions because of the confinement effect of their featured cages^41^,^42^,^43^.

Due to these important applications, speculating how many unknown ABC-6 structures are realizable as new catalysts with desired properties is of great significance for the development of such materials. However, to answer this question is challenging. First, we need a highly efficient computational method to enumerate all possible ABC-6 structures. Second, we need to evaluate all enumerated structures and remove the unrealizable ones. More importantly yet more difficultly, we need a high-throughput structure screening method to identify candidate ABC-6 structures with desired properties according to functional needs. An early attempt was made towards answering this question, but failed in structure evaluation and structure identification^44^.

Here we propose a new genomic approach towards the solution of these problems. In this work, we focus on the one-dimensional digital stacking sequences, that is, the genes of ABC-6 structures. By enumerating all possible stacking sequences, we are able to predict every ABC-6 topology that is chemically feasible. We have developed a ternary numeral coding system, in which each stacking sequence is expressed as a specific ternary numeral. To enumerate all possible stacking sequences, we went through all ternary numerals from the smallest one to the largest allowed and evaluated the chemical feasibility for each one of them. During this enumeration process, equivalent stacking sequences (for instance, (BCA), (CAB), (CBA), (ACB) and (ABCABC), and so on, are all equivalent to (ABC)) and chemically infeasible ones (for instance, (AAA) is chemically infeasible because each stacking layer in it is highly distorted from the ideal tetrahedral coordination) were removed. At the end of the enumeration, every one of our saved stacking sequences corresponded to a topologically unique and chemically feasible ABC-6 topology.

Besides structure enumeration, our genomic approach provides a high-throughput way to extract the most important structural information directly from the enumerated stacking sequences. For instance, our computer program can locate all constituent cages hidden in the stacking sequences, which are the most important structural features for ABC-6 zeolites. To do this, our computer program went through the corresponding stacking sequence back and forth to look for a string of any length that could be interpreted as a valid ABC-6 cage. Such a string should start and end with the same letter, and this letter should not appear in the middle of this string. By finding all such strings in a stacking sequence, we have located all constituent cages in every enumerated ABC-6 topology (Fig. 1). Besides constituent cages, some other structural features, such as the channels and the stacking compactness of six-ring layers are also important to ABC-6 zeolites. Channels link up ABC-6 cages to form a three-dimensional porous system, so their widths and orientations are crucial to the adsorption and diffusion of guest species. Some ABC-6 structures may possess narrow channels only, the openings of which are no wider than a six-ring; other structures may possess interconnecting 8-ring channels perpendicular to the *c*-axis or/and 12-ring channels running along the *c*-axis. Besides cages and channels, how compactly the six-ring layers are stacked is another important structural feature influencing the porosity and other related properties of ABC-6 zeolites. Highly compact stacking of six-ring layers leads to dense ABC-6 frameworks, whereas less compact stacking gives rise to frameworks with more accessible void spaces for guest species, which are highly desired for many applications. Because of the intrinsic nature of ABC-6 structures, compact stackings only occur between successive distinct layers, and those between successive identical layers are non-compact stackings. Here we define, for the first time, the stacking compactness of an ABC-6 structure as the difference in the numbers of compact and non-compact stackings divided by the total number of layer stackings. According to this definition, the highest stacking compactness of an ABC-6 structure is 1, corresponding to the densest framework where all layers are compactly stacked. The lowest stacking compactness is 0, corresponding to the most porous framework where only half of the layer stackings are compact. Via high-throughput interpretation of the stacking sequences, the information on channels and stacking compactness can be extracted by our computer program. Details regarding the enumeration and interpretation of ABC-6 stacking sequences can be found in the Methods section.

## Results

### Enumeration of ABC-6 structures

Considering the computational cost, we have enumerated 84,292 stacking sequences corresponding to all topologically unique and chemically feasible ABC-6 topologies comprised of *N* stacking layers (*N*≤16). The results are summarized in Supplementary Table 2. In all, 98.8% of the enumerated ABC-6 topologies possess 8-ring channels, far outnumbering the ones with 12-ring channels (0.2%) and the ones with 6-ring channels only (1.1%). The distribution of ABC-6 topologies among seven possible symmetries is also uneven. 95.7% of the ABC-6 topologies have the symmetry of *P*3*m*1, 2.3 and 1.7% belong to *P*-3*m*1 and *P*-6*m*2, respectively, and those belonging to other symmetries amount only to 0.3%. Most of the enumerated ABC-6 topologies consist of 5∼9 types of constituent cages.

**Figure 2:**
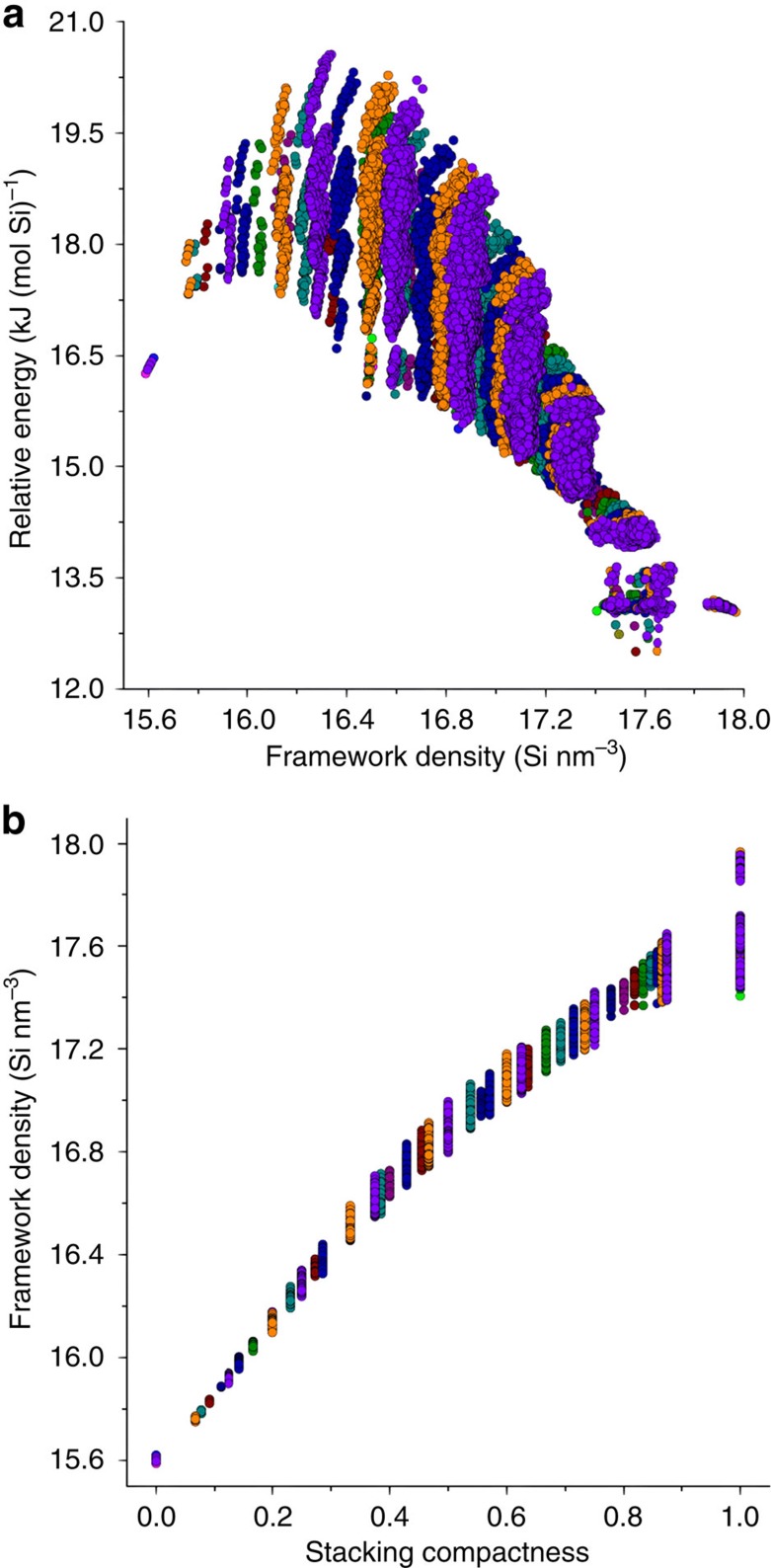
Structural attributes of 84,292 optimized ABC-6 models. (**a**) Framework energy versus framework density. The ranges of framework energies and framework densities are both consistent with those of existing ABC-6 zeolites. (**b**) Framework density versus stacking compactness. Stacking compactness reflects how compactly the six-ring layers are stacked in an ABC-6 structure, which is obviously proportional to framework density. ABC-6 models comprised of different numbers of stacking layers are shown in different colours in these two plots.

From the stacking sequences we enumerated, we built the corresponding 84,292 atomic models. All of these models were fully optimized as silica polymorphs through a classic molecular mechanics method (see the Methods section and our online database^45^ for more details). The framework energies relative to quartz for all of these models vary between 12.5 and 20.6 kJ (mol Si)^−1^, and the framework densities vary between 15.6 and 18.7 Si nm^−3^ (Fig. 2a), agreeing well with those of existing ABC-6 zeolites. Moreover, statistics on Si–O, O–Si–O, and Si–O–Si distances in these models well obey the local interatomic distances (LIDs) criteria^19^ recently discovered among all existing zeolites, indicating that all of our enumerated topologies are chemically feasible as tectosilicates (see the Methods section for more details). Figure 2b plots the framework density versus stacking compactness for 84,292 optimized ABC-6 models. The stacking compactness is proportional to framework density, just as it is defined. According to this plot, we are able to estimate the framework density of an unknown ABC-6 structure directly from its corresponding stacking sequence.

### Grouping of ABC-6 structures

**Figure 3:**
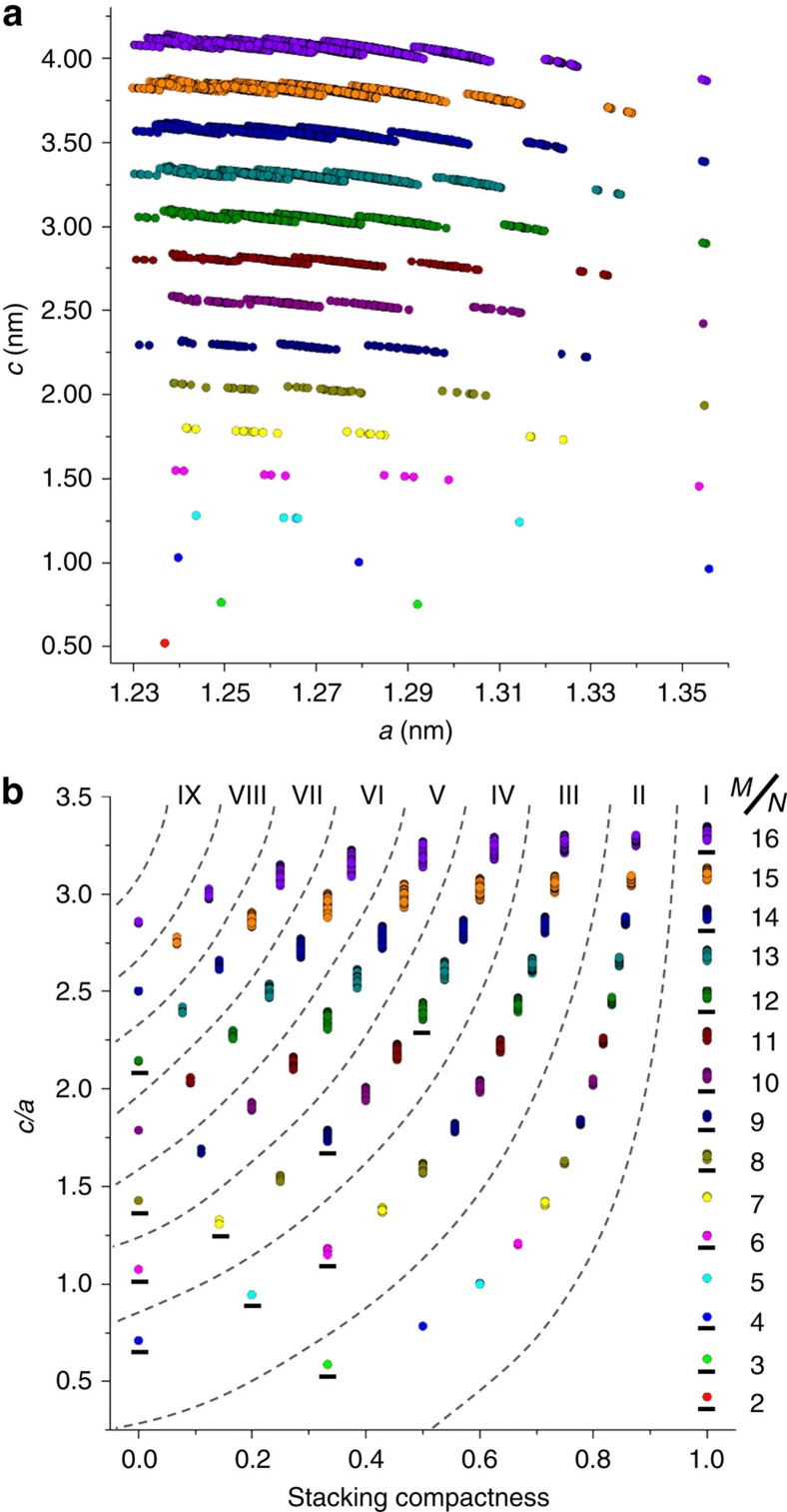
Grouping of 84,292 optimized ABC-6 models. (**a**) Plot of lattice dimensions *c* versus *a*. (**b**) Plot of *c*/*a* versus stacking compactness. Each individual group can be named according to the number of stacking layers (*N*) and the rank of its corresponding stacking compactness (*M*). Groups having at least one end member realized already are underlined with short bars. ABC-6 models comprised of different numbers of stacking layers are shown in different colours in these two plots.

Figure 3a demonstrates the plot of lattice dimensions *c* versus *a* for all of the optimized atomic models. The *a* dimensions of the optimized models vary between 1.230 and 1.356 nm, and the *c* dimensions vary between 0.241 × *N* and 0.259 × *N* nm, where *N* is the number of stacking layers. Surprisingly, all of these models seem to cluster into several groups even for those with identical *N*. We believe that the grouping of ABC-6 models should arise from the discreteness of their stacking compactness values. For *N*-layered ABC-6 topologies, the stacking compactness may have *N*/2+1 or (*N*-1)/2+1 possible values, depending on whether *N* is an even or odd number. Figure 3b is the plot of *c*/*a* versus stacking compactness, showing the perfect grouping of 84,292 optimized ABC-6 models according to *N* and the stacking compactness. Thus, all ABC-6 topologies comprised of ≤16 stacking layers can be divided into 78 groups, 20 of which have at least one end member realized already (underlined with short bars in Fig. 3b).We can name each individual group in the form of *N*–*M*, where *M* (written as a Roman numeral) is the rank of its corresponding stacking compactness among all possible values for *N*-layered structures. For instance, six-layered ABC-6 structures may have four possible stacking compactness values, that is, 6/6, 4/6, 2/6, and 0/6. Thus, among all 6-layered structures, liottite ((ABABAC)) with the highest stacking compactness of 6/6 belongs to Group 6-I, erionite ((AABAAC)) and bellbergite ((AABCCB)) with a stacking compactness of 2/6 belong to Group 6-III, and chabazite ((AABBCC)) with the lowest stacking compactness of 0/6 belongs to Group 6-IV. Figure 3 can be used as a reference to determine the framework structures of new ABC-6 zeolites. When the lattice dimensions of a new ABC-6 zeolite are known, we may refer to these plots to determine which groups the new structure may belong to. Then, the most probable atomic models will be determined from these groups by examining whether their simulated X-ray diffraction patterns match the observed one.

### Identification of the most realizable ABC-6 topologies

**Figure 4:**
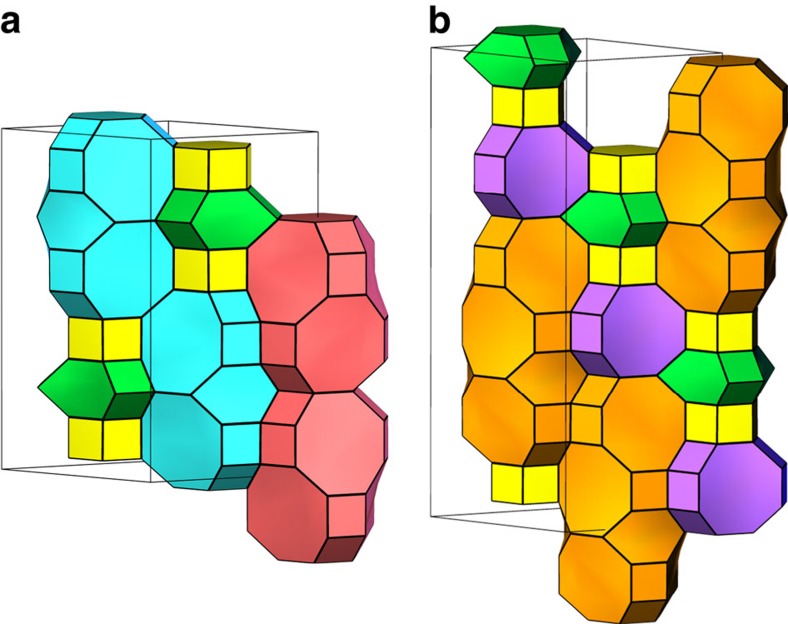
Two new ABC-6 topologies recently realized by the authors. (**a**) Magnesium aluminophosphate JU-60 ((AABAACCBCC)) is constructed by four types of ABC-6 cages, being the first member in Group 10-V. (**b**) Zinc aluminophosphate JU-61((AABAABBCBBCCACC)) is the first member in Group 15-VII. The framework of JU-61 is also constructed from four types of ABC-6 cages, and one of them ((abbcbbcca)) has never been observed in any existing ABC-6 zeolites. Different types of ABC-6 cages are shown in different colours in this figure.

Although all of our enumerated ABC-6 topologies are chemically feasible as tectosilicates, only 23 of them have been realized as natural minerals or synthetic materials. Among these realized ABC-6 topologies, half possess six-ring channels only, contradicting the enumeration result that only 1.1% of the enumerated topologies do (Supplementary Table 2). We believe these contradictions arise from the fact that many of our enumerated topologies are not practically realizable. Thus far, we have only considered the chemical feasibility of the host frameworks, yet neglecting the contribution of extra-framework cations, anion groups, or water molecules inside the ABC-6 cages. As a matter of fact, all of the realized ABC-6 frameworks can only form when extra-framework species are present, implying that they are highly important to the formation of ABC-6 structures. Considering the strong host-guest interactions between ABC-6 cages and extra-framework species, we believe that these featured constituent cages may hold the key to improve our prediction. After careful examination of the structural information we have extracted from the stacking sequences, we determine, for the first time, that all realized ABC-6 topologies are comprised of no more than four types of constituent cages, as is the case even for 36-layered kircherite, the most complex ABC-6 zeolite ever (Supplementary Table 1). This phenomenon is reasonable because every type of ABC-6 cage holds a specific collection of extra-framework species, which can form only under specific reaction conditions. Structures comprised of many types of cages can form only when the reaction conditions for all constituent cages are simultaneously fulfilled, which will be too difficult to occur in reality. Among the 23 already-realized ABC-6 topologies with ≤16 stacking layers, 2 are comprised of 1 type of cages, 6 comprised of 2, 14 comprised of 3, and the remaining 1 comprised of 4. In contrast, nearly 99% of the enumerated ABC-6 topologies are comprised of 5∼9 types of constituent cages (Supplementary Table 2). After removing all enumerated structures that are comprised of more than four types of cages, only 1,150 remained in the end (Table 1 and Supplementary Fig. 1; see our online database^45^ for more details). Half of these 1,150 topologies possess six-ring channels only, which is consistent with the situation of realized ABC-6 zeolites. The cell dimensions, space groups, largest channel openings, framework energies, framework densities, stacking compactness and extracted constituent cages for these 1,150 ABC-6 structures are provided in Supplementary Data 1. In addition, we have calculated the theoretical solvent-accessible pore volumes and surface areas with respective to H_2_O, H_2_, CO_2_, N_2_, and CH_4_ for these ABC-6 structures (Supplementary Data 1; see the Methods section for more details). The fractional pore volumes for these five important probe molecules are in the ranges of 5.24–11.93%, 4.05–9.91%, 2.48–7.40%, 1.55–5.75% and 1.21–5.09%, respectively, and the surface areas are in the ranges of 5.55–11.30 Å^2^ Si^−1^, 4.66–10.07 Å^2^ Si^−1^, 3.41–7.62 Å^2^ Si^−1^, 2.54–6.03 Å^2^ Si^−1^ and 2.17–5.39 Å^2^ Si^−1^, respectively. These data can be used to prescreen candidate structures for specific gas adsorption or separation applications. Among the 1,150 ABC-6 topologies constructed by no more than four types of constituent cages, 23 have already been realized. We deem the remaining 1,127 ABC-6 structures the most realizable synthetic candidates, because they are both chemically feasible and practically easy to form together with extraframework species. Recently, we have successfully realized two of these candidates, that is, magnesium aluminophosphate JU-60 and zinc aluminophosphate JU-61. These two new ABC-6 zeolites were both synthesized using 1,2-diaminocyclohexane as the structure-directing agent under hydrothermal conditions, and both of their structures were determined through single-crystal X-ray diffraction (see the Methods section for more details about their synthesis and structure determination). JU-60 belongs to Group 10-V, and its corresponding stacking sequence is (AABAACCBCC). JU-60 is comprised of four types of cages, including hexagonal prisms ((aa)), cancrinite cages ((aba)), chabazite cages ((abbcca)) and erionite cages ((abbcbba)), respectively (Fig. 4a). JU-61 belongs to Group 15-VII, and it is the first ABC-6 zeolite comprised of 15 stacking layers ((AABAABBCBBCCACC)). JU-61 consists of four types of cages, including the hexagonal prisms, cancrinite cages, gmelinite cages ((abba)), and a new type of ABC-6 cage ((abbcbbcca)), respectively (Fig. 4b). The synthesis of these new ABC-6 zeolites once again validates our prediction of the most realizable ABC-6 topologies.

## Discussion

**Figure 5:**
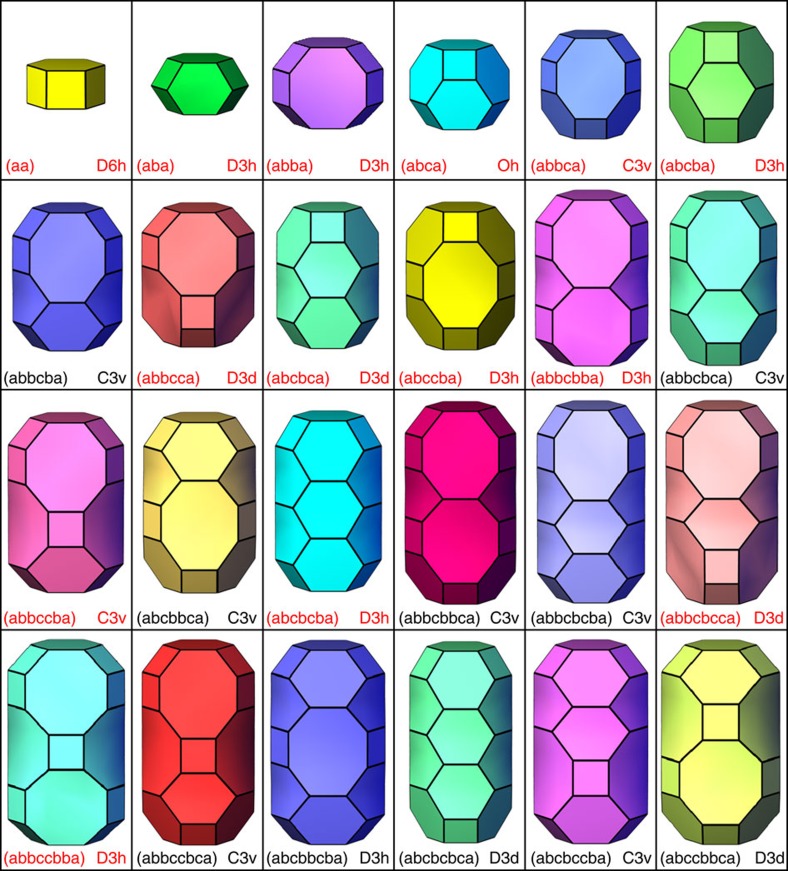
Enumerated ABC-6 cages constructed by ≤8 six-ring layers. For each cage, the stacking sequence and the highest allowed symmetry are given. The stacking sequences representing the cages that have already been observed in existing ABC-6 materials are highlighted in red.

Focusing on the stacking sequences of ABC-6 zeolites, our genomic approach has provided a straightforward and reliable way to predict the most realizable synthetic candidates. More importantly, the key structural information, especially regarding the constituent cages, can be directly extracted from these stacking sequences, the genes of ABC-6 zeolites. Through a computer procedure similar to the enumeration of ABC-6 structures, we have enumerated 57 types of ABC-6 cages comprised of no more than 10 six-ring layers (Fig. 5 and Supplementary Fig. 3; see the Methods section for more details). As the physical and chemical properties of ABC-6 zeolites are mainly determined by their constituent cages, examining these ABC-6 cages enables the high-throughput screening of ABC-6 zeolites for specific applications. For instance, methanol-to-olefin (MTO) conversion over acidic zeolite catalysts has been an important non-petrochemical industrial process to produce highly demanded light olefins via natural gas, coal, or even biomass^46^,^47^. Chabazite and its synthetic counterparts are currently among the best catalysts for MTO reactions, and the shape and size of their featured cage ((abbcca)) are believed to play the key role in this type of reactions by providing suitable confined void space^43^. To find new ABC-6 catalysts with better MTO performance than chabazite, we have performed density functional theory (DFT) calculations on the methylation of hexamethylbenzene within different ABC-6 cages assuming the same ‘hydrocarbon pool' mechanism^48^,^49^. This reaction occurs at the beginning of an MTO process and is believed to be the key step to initiate the MTO process^50^. We have calculated seven ABC-6 cages that are in similar size to the chabazite cage and possess many eight-ring windows in favour of olefin diffusion. The reaction barriers and reaction energies of these ABC-6 cages, as well as those of the chabazite cage, are listed in Supplementary Table 9. Two of these ABC-6 cages ((abbccbba) and (abbccbca)) exhibit significantly lower reaction barriers and reaction energies than the chabazite cage, indicating that they may provide more suitable confinement effect on the hydrocarbon species than the chabazite cage (Supplementary Fig. 4). By checking the stacking sequences of the 1,127 most realizable synthetic candidates, we have found that only seven of them possess these ‘superior' cages (Supplementary Data 1). In particular, two of these seven ABC-6 structures ((AABBAACCAABBCC) and (AABBAACCBBAABBCC)) possess large accessible pore volumes comparable to chabazite, making them the most promising synthetic candidates as new MTO catalysts.

Notably, we assume all enumerated ABC-6 topologies are silicate zeolites in this work. In fact, these topologies may also be realizable as other tetrahedrally coordinated materials, such as silicon sulfides, alkali halides, sp^3^ carbon or silicon allotropes, and Zintl phases, which may have interesting mechanical, electronic, optical and chemical properties^51^,^52^. In particular, a series of zeolitic imidazolate frameworks with ABC-6 topologies have been reported recently, which exhibit the highly desired capability for the capture of fission product^53^ and CO_2_ (ref. 54). Moreover, our genomic approach is valid not only for ABC-6 structures but also for other crystalline materials that are constructed from the stacking of well-defined modular layers.

## Methods

### Enumeration of ABC-6 stacking sequences

Our computer programs for the enumeration and interpretation of ABC-6 stacking sequences were written in FORTRAN. To enumerate all possible stacking sequences of length *N*, our computer program went through every *N*-digit ternary numeral from the smallest one to the largest allowed. Because only unique stacking sequences were needed, we fixed the first digit of every ternary numeral to be ‘0' and enumerated the remaining (*N*−1) digits. To guarantee that only the chemically feasible and topologically unique stacking sequences were retained, our computer program performed a two-step examination procedure for each numeral visited. First, our program checked if the current numeral consisted of three or more successive identical digits. If not, this numeral should represent a chemically feasible stacking sequence. Then, our program generated all equivalent numerals for the current one and examined whether any of these equivalent numerals was smaller than the current one. If not, then the current numeral should represent a new stacking sequence. Only the ternary numerals passing both examinations were saved by our computer program. This examination procedure was repeated until the largest allowed *N*-digit numeral was achieved. The enumeration of ABC-6 cages followed a similar procedure. The only difference was that the ternary numeral for a valid ABC-6 cage should start and end with the same digit, and this digit must be absent in the middle of this ternary numeral. To ensure that all our enumerated cages are topologically unique, we fixed the starting and ending digits to be ‘0' and enumerated the middle part with digits ‘1' and ‘2' only.

### Structural information extraction

To extract the channel information from the stacking sequences, our computer program checked the following situations: (1) if a stacking sequence was comprised of only two types of letters, it represented an ABC-6 topology with 12-ring channels running along the *c*-direction; (2) if a stacking sequence consisted of successive identical letters, it indicated the existence of interconnecting 8-ring channels perpendicular to the *c*-direction; (3) other stacking sequences corresponded to ABC-6 topologies with six-ring channels only. To calculate the stacking compactness of an ABC-6 topology from its stacking sequence, our computer program counted the number of letters that were distinct from both of their neighbours and divided that value by *N*, the number of stacking layers.

### Geometry optimization of ABC-6 models

The atomic models for 84,292 enumerated stacking sequences were built as silica polymorphs using Materials Studio (Accelrys Software Inc., 2005). The highest symmetries of these models were identified by the ‘Find Symmetry' tool implemented in Materials Studio. These models were fully optimized without symmetry constraints by GULP^55^ with the Sanders-Leslie-Catlow potentials^56^. All structural models were confirmed to have no imaginary phonon mode.

### Evaluation of the optimized ABC-6 models

To evaluate the chemical feasibility of the optimized ABC-6 structures, we have also optimized 208 existing zeolites known to date^57^ as silica polymorphs using the same empirical potentials. The framework densities and framework energies of our enumerated ABC-6 structures agreed well with those of 208 existing zeolite structures. Recently, we proposed a set of LIDs criteria^19^, which have proved to be more effective and reliable for structure evaluation than other methods. According to these criteria, the means, standard deviations, and ranges of LIDs in a chemically feasible zeolite structure, including T–O, O–T–O, and T–O–T distances, should obey a set of relationships. In this work, the LIDs in 84,292 optimized ABC-6 structures were calculated using the program FraGen^58^, and the results showed that all of these enumerated ABC-6 structures were chemically feasible. The LIDs for 1,150 most realizable ABC-6 structures are provided as Supplementary Data 2. The solvent-accessible pore volumes and surface areas for 1,150 most realizable ABC-6 structures were calculated using the ‘Volume' tool implemented in Materials Studio. Rigid spheres with diameters of 2.65, 2.89, 3.30, 3.64 and 3.80 Å were used as the probes, corresponding to the kinetic diameters of H_2_O, H_2_, CO_2_, N_2_ and CH_4_, respectively.

### Synthesis of JU-60 and JU-61

Magnesium aluminophosphate JU-60 and zinc aluminophosphate JU-61 were both synthesized using 1,2-diaminocyclohexane (DACH) as the structure-directing agent under hydrothermal conditions. To synthesize JU-60, 0.1 g of pseudoboehmite (Al_2_O_3_, 74.3%) and 0.3 g of magnesium acetate were dispersed in 10 ml of H_2_O with stirring for 2 h. A volume of 0.5 ml of DACH (99 wt %) was then added into the mixture with stirring, followed by the addition of 0.2 ml of H_3_PO_4_ (85 wt %). A homogeneous gel was formed with an overall molar composition of 1.0 MgO: 0.5 Al_2_O_3_: 2.1 H_3_PO_4_: 2.9 DACH: 404 H_2_O. The gel was transferred into a 15-ml Teflon-lined stainless steel autoclave and heated at 180 °C for 3 days. The obtained crystals of JU-60 were separated by filtration, washed with distilled water and dried in air at room temperature. To synthesize JU-61, 0.25 g of pseudoboehmite (Al_2_O_3_, 62.5%) was dispersed in a mixture of 8 ml of H_2_O and 0.4 ml of H_3_PO_4_ (85 wt%), followed by the addition of 0.41 g of ZnCl_2_. After stirring for 2 h, 1.5 ml of DACH (99 wt%) was added. A homogeneous gel was formed after stirring for another 2 h, with an overall molar composition of 1.0 ZnO: 0.5 Al_2_O_3_: 2.0 H_3_PO_4_: 4.0 DACH: 152 H_2_O. The gel was transferred into a 15-ml Teflon-lined stainless steel autoclave and heated at 180 °C for 5 days. The obtained crystals of JU-61 were separated by filtration, washed with distilled water and dried in air at room temperature.

### X-ray structure determination for JU-60 and JU-61

Powder X-ray diffraction data were collected on a Rigaku D/max-2550 diffractometer with Cu Kα radiation (*λ*=1.5418 Å). Single-crystal X-ray diffraction data were collected on a Bruker AXS SMART APEX II diffractometer using graphite-monochromated Mo Kα radiation (*λ*=0.71073 Å) at the temperature of 23±2 °C. Data processing was accomplished with the SAINT processing program. The framework structures of JU-60 and JU-61 were solved by direct methods and refined on *F*^2^ by full matrix least-squares techniques with SHELXTL. Parts of the extra-framework species, such as DACH and water molecules, were located during least-squares refinement. JU-60 consists of three crystallographically distinct Al and three P sites. One of the Al site exhibited an average Al-O bond distance of 1.85 Å, indicating that it was half occupied by Mg. JU-61 consisted of five crystallographically distinct tetrahedrally coordinated sites. Considering the restrictions of the odd number of layers and the Loewenstein's rule^59^, we had to refine the structure of JU-61 assuming that all of the five tetrahedrally coordinated sites were co-occupied by disordered Al, P, and Zn. The occupancy ratio of Zn to Al was fixed to 2:3 according to the average bond distance in JU-61 (1.66 Å). To remove these disorders, we have also made several attempts to index JU-61 in a doubled unit cell, but the data collected in this way were not good enough for a feasible structure solution. The crystallographic tables, atomic coordinates, selected bond distances and angles, and powder X-ray diffraction patterns for JU-60 and JU-61 are provided in Supplementary Tables 3–8 and Supplementary Fig. 2.

### Density functional theory calculations

All of the cage models were cut from the optimized ABC-6 structures. For each ABC-6 cage, one of the Si atoms in the eight-ring window was replaced by Al to produce the Brönsted acid site. The dangling bonds in all cages were saturated by H atoms. All atoms in ABC-6 cages and extra-framework species were fully optimized without any constraint at ONIOM(B3LYP/6–31G(d,p):AM1) level^60^,^61^,^62^, where the acid site (SiO_3_–O–AlO_2_–OH–SiO_3_ cluster) and extraframework species were in the high level (Supplementary Fig. 4). The achievement of energy minima or saddle points was checked by frequency calculations at the same level. The reaction barrier was calculated as the energy difference between the transition state and the reactant (hexamethylbenzene, methanol and the protonated ABC-6 cage). The reaction energies were calculated as the energy differences between the product (heptamethylbenzenium cation, water, and the deprotonated ABC-6 cage) and the reactant. To improve the precision of weak interaction energy calculations, we have performed single-point energy calculations at ωB97XD/6–31+G(d,p) level^63^ for all optimized models. All density functional theory calculations were carried out using the Gaussian 09 package^64^.

## Additional information

**How to cite this article:** Li, Y. *et al.* *In silico* prediction and screening of modular crystal structures via a high-throughput genomic approach. *Nat. Commun.* 6:8328 doi: 10.1038/ncomms9328 (2015).

## Supplementary Material

Supplementary Information Supplementary Figures 1-4, Supplementary Tables 1-9

Supplementary Data 1 1,150 enumerated ABC-6 structural models constructed from no more than four types of cages (including known ABC-6 types).

Supplementary Data 2 The means (<D>), standard deviations (σ), and ranges (R) of the local Interatomic Distances (T-O, O-O, and T-T) in 1,150 enumerated ABC-6 structures with no more than four types of constituent cages.

## Figures and Tables

**Figure 1:**
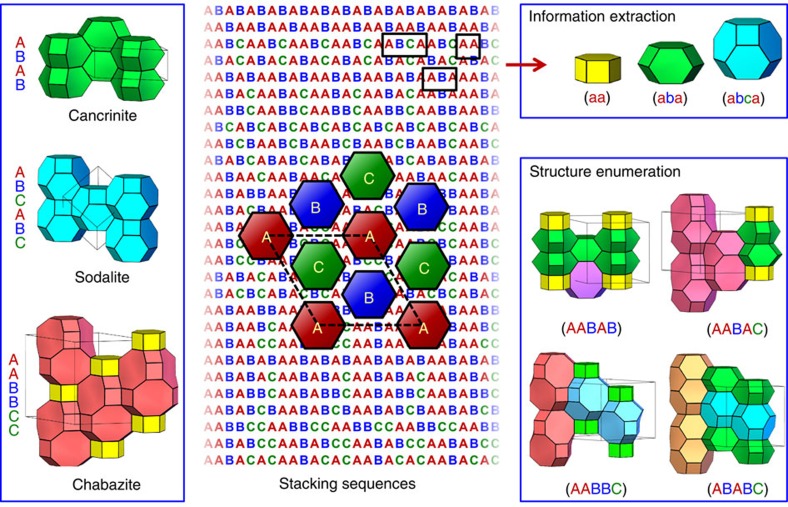
Enumeration and interpretation of ABC-6 stacking sequences. All ABC-6 topologies are constructed from the stacking of three types of 6-ring layers (denoted by A, B, and C, respectively) along the *c*-direction in hexagonal unit cells. The sequences of the stacking of these modular layers determine the entire structures as well as the physical and chemical properties of ABC-6 zeolites, so we deem them the genes of this family. By enumerating every possible ternary stacking sequence, we are able to predict the structures of all topologically unique and chemically feasible ABC-6 structures. More importantly, lots of structural information, especially regarding the constituent cages that are crucial to the property and realizability of ABC-6 topologies, can be extracted directly from these stacking sequences. This figure shows the structures of cancrinite, sodalite and chabazite (left), the schematic drawing of the three types of six-ring layers with some of the enumerated stacking sequences (middle), some constituent cages determined from the stacking sequences (top right), and four enumerated ABC-6 structures comprised of five stacking layers (bottom right).

**Table 1:** Numbers of topologically unique and practically realizable ABC-6 topologies*.

**Number of constituent layers**	**Largest channel opening**			**Total**
	**6-ring**	**8-ring**	**12-ring**	
2	0	0	1(1)	1(1)
3	1(1)	0	1(1)	2(2)
4	1(1)	1	1(1)	3(2)
5	1	2(1)	1	4(1)
6	2(1)	4(3)	1	7(4)
7	3	5(1)	2	10(1)
8	6(1)	11(1)	3	20(2)
9	7(1)	10(1)	3	20(2)
10	15(2)	20	6	41(2)
11	18	22	7	47
12	39(2)	35(2)	11	85(4)
13	46	41	14	101
14	100(1)	63	24	187(1)
15	130	57	28	215
16	254(1)	104	49	407(1)
Total	623(11)	375(9)	152(3)	1150(23)

^*^The numbers in brackets are the numbers of topologies that have already been realized.
