# Supplementary material for: In silico prediction and screening of modular crystal structures via a high-throughput genomic approach
Source: Nat Commun. 2015 Sep 23;6:8328. doi: 10.1038/ncomms9328 (PMC4667440; doi:10.1038/ncomms9328)
Supplement: Supplementary Information — Supplementary Figures 1-4, Supplementary Tables 1-9 [file ncomms9328-s1.pdf]

Supplementary Table 1 | Realized ABC-6 topologies grouped by the number of stacking layers*.

Staking Layers	Stacking sequence	Material names	Constituent cages
2	(AB)	balliranoite; cancrinite; cancrisilite; davyne; depmeierite; hydroxycancrinite; kyanoxalite; microsommite; quadridavyne; pitiglianoite; tiptopite; vishnevite	(aba)
3	(AAB)	offretite; LZ-217#; RMA-4#; TMA-O#	(aa); (aba); (abba)
	(ABC)	bicchulite; danalite; genthelvite; haüyne; helvine; kamaishilite; lazurite; nosean; sodalite; tsaregorodsevite; tugtupite; AlPO-20#; SIZ-9#; sodalite-AlGe#; sodalite-BeP#; sodalite-GaGe#	(abca)
4	(AABB)	gmelinite-Ca; gmelinite-K; gmelinite-Na; gmelinite-BeP#	(aa); (abba)
	(ABAC)	bystrite; carbobystrite; losod#; losod-AlGe#; losod-BeP#	(aba); (abcba)
5	(AABBC)	ZnAlPO-57#; SAPO-57#	(aa); (abbca); (abbcca)
6	(AABAAC)	erionite-Ca; erionite-K; erionite-Na; AlPO-17#; LZ-220#; UZM-12#	(aa); (aba); (abbcbba)
	(AABBCC)	chabazite-Ca; chabazite-K; chabazite-Mg; chabazite-Na; chabazite-Sr; willhendersonite; DAF-5#; SAPO-34#; SSZ-13#; UiO-21#	(aa); (abbcca)
	(AABCCB)	bellbergite; TMA-E#	(aa); (abba); (abccba)
	(ABABAC)	liottite	(aba); (abcba); (abcbcba)
7	(AABBACC)	ZnAlPO-59#; SAPO-59#	(aa); (abba); (abbccba)
8	(AABBAACC)	SAPO-56#; SSZ-16#	(aa); (abba); (abbccbba)
	(ABABACAC)	afghanite; alloriite	(aba); (abcbcba)
9	(AABCCABBC)	levyne-Ca; levyne-Na; SAPO-35#; SAPO-67#	(aa); (abbca)
	(ABABCBCAC)	levyne-b#	(aba); (abcbca)
10	(ABACABCABC)	franzinite	(aba); (abca); (abcba)
	(ABABABACAC)	franzinite-b#	(aba); (abcbcba); (abcbcbcba)
12	(AABABBCBCCAC)	STA-2#	(aa); (aba); (abbcbcca)
	(AABBAACCBBCC)	AlPO-52#	(aa); (abba); (abbcca);

Staking Layers	Stacking sequence	Material names	Constituent cages
			(abbccbba)
	(ABABACBABABC)	marinellite	(aba); (abca); (abcbcba)
	(ABABACABACAC)	tounkite	(aba); (abcba); (abcbcba)
14	(ABABACBACACABC)	farneseite	(aba); (abca); (abcbcba)
16	(ABABABACBABABABC)	giuseppettite	(aba); (abca); (abcbcbcba)
18	(AABBAABBCCBBCCAACC)	SSZ-52#	(aa); (abba); (abbccbbcca)
28	(ABABACABCABCABACACABACBACBAC)	sacrofanite	(aba); (abca); (abcba); (abcbcba)
30	(ABABACBACBACABACBACBACBCABCABC)	biachellaite	(aba); (abca); (abcba); (abcbcba)
33	(ABACABCABCABCBABCABCABCACBCABCABC)	fantappieite	(aba); (abca); (abcba)
36	(ABACABCABCABCACBCABCABCABCBABCABCABC)	kircherite	(aba); (abca); (abcba)

*For each realized ABC-6 topology, the corresponding stacking sequence, the names of natural and synthetic (marked by '#') zeolites taking on the corresponding topology and the stacking sequences of constituent cages are given. Data in this table were obtained from IMA Database of Mineral Properties (http://rruff.info/ima/) and Database of Zeolite Frameworks (http://www.iza-structure.org/databases/).

Supplementary Table 2 | Numbers of topologically unique and chemically feasible ABC-6 topologies grouped by the number of staking layers, the largest channel opening, the highest allowed symmetry, and the number of constituent cage types.

Staking layers	Channel opening			Space group							Number of constituent cage types										Total
	6-ring	8-ring	12-ring	*P*3*m*1	*R*3*m*	*P*-3*m*1	*R*-3*m*	$P6_3mc$	*P*-6*m*2	$P6_3/mmc$	1	2	3	4	5	6	7	8	9	10	
**2**			1							1	1										1
**3**	1		1				1		1		1		1								2
**4**	1	1	1			1				2		2	1								3
**5**	1	3	1			3			2				4		1						5
**6**	2	7	1	3		2	1		1	3		1	5	1	3						10
**7**	3	15	2	7		7			6				7	3	6	4					20
**8**	6	36	3	21		11		1	7	5		1	8	11	17	6	2				45
**9**	7	86	3	62		17	2		15			2	6	12	41	28	7				96
**10**	16	208	6	158		37		3	23	9			20	21	77	74	36	2			230
**11**	21	501	7	430		55			44				19	28	135	210	121	16			529
**12**	43	1249	11	1100	1	106	2	11	71	12		1	27	57	278	418	434	88			1303
**13**	63	3086	14	2894		149			120				32	69	429	1020	1187	416	10		3163
**14**	129	7815	24	7398		312		27	204	27			59	128	795	2053	3210	1625	98		7968
**15**	203	19727	28	19226	3	400	4		325			1	62	152	1342	3997	8246	5428	726	4	19958
**16**	404	50506	49	49385		876		78	582	38		1	99	307	2314	8006	19359	17056	3755	62	50959
**Total**	900	83240	152	80684	4	1976	10	120	1401	97	2	9	350	789	5438	15816	32602	24631	4589	66	84292

Supplementary Table 3 | Crystal data and structure refinement details for JU-60.

Identification code	JU-60
Empirical formula	C1.50 Al2 Mg0.50 O10.33 P2.50
Formula weight	326.89
Temperature	273(2) K
Wavelength	0.71073 Å
Crystal system	Hexagonal
Space group	$P6_3/m$
Unit cell dimensions	a = 13.466(3) Å α = 90°.
	b = 13.466(3) Å β = 90°.
	c = 25.758(6) Å γ = 120°.
Volume	4045(2) $Å^3$
Z	12
Density (calculated)	1.610 Mg/m^3
Absorption coefficient	0.569 mm^{-1}
F(000)	1934
Theta range for data collection	1.581 to 25.348°.
Index ranges	$-16 \le h \le 15, -15 \le k \le 16, -26 \le l \le 31$
Reflections collected	43324
Independent reflections	2531 (R(int) = 0.0688)
Completeness to theta = 25.242°	100.0 %
Refinement method	Full-matrix least-squares on F^2
Data / restraints / parameters	2531 / 0 / 167
Goodness-of-fit on F^2	1.223
Final R indices (I > 2sigma(I)) *	$R1$ = 0.0908, $wR2$ = 0.2506
R indices (all data) *	$R1$ = 0.1073, $wR2$ = 0.2685
Largest diff. peak and hole	1.366 and −0.579 e.$Å^{-3}$

* $w = 1/(\sigma^2(Fo^2) + (0.1317P)^2 + 17.7566P)$ where $P = (Fo^2 + 2Fc^2)/3$

Supplementary Table 4 | Atomic Coordinates (× 10^4) and equivalent isotropic displacement parameters (Å^2 × 10^2) for JU-60.

Atom	*x*	*y*	*z*	*U*(eq)
P(1)	8998(2)	-4288(2)	5383(1)	26(1)
P(2)	6649(2)	-4306(2)	6637(1)	24(1)
P(3)	10080(3)	-2352(3)	7500	26(1)
Mg/Al(1)	9058(2)	-4253(2)	6613(1)	28(1)
Al(2)	6620(2)	-4377(2)	5426(1)	24(1)
Al(3)	7658(3)	-2443(3)	7500	23(1)
O(1)	7553(7)	-4633(6)	6734(3)	49(2)
O(2)	9882(9)	-3555(7)	4979(3)	58(2)
O(3)	10035(6)	-3014(6)	7015(3)	51(2)
O(4)	6918(7)	-3246(7)	6949(2)	48(2)
O(5)	5500(6)	-5288(7)	6785(2)	49(2)
O(6)	11182(9)	-1237(9)	7500	44(2)
O(7)	8692(6)	-5529(7)	5323(3)	50(2)
O(8)	9436(7)	-3843(7)	5915(2)	49(2)
O(9)	6631(7)	-3982(6)	6074(2)	42(2)
O(10)	7948(7)	-4204(6)	5260(2)	49(2)
O(11)	9100(9)	-2106(8)	7500	45(2)
O(1W)	6667	-6667	7500	68(5)
O(2W)	4470(50)	-4200(50)	7500	89(14)
C(1)	9370(40)	-1230(30)	5977(11)	59(7)
C(2)	10640(40)	-610(40)	6073(12)	72(9)
C(3)	7290(50)	-5440(30)	3965(14)	79(9)
C(4)	7810(40)	-6110(60)	3919(18)	112(14)

Supplementary Table 5 | Selected bond distances (Å) and angles (°) for JU-60.

P(1)-O(8)	1.495(7)	O(4)-P(2)-O(9)	105.4(4)
P(1)-O(10)	1.507(8)	O(6)-P(3)-O(11)	108.5(5)
P(1)-O(7)	1.515(8)	O(6)-P(3)-O(3)	108.5(4)
P(1)-O(2)	1.516(8)	O(11)-P(3)-O(3)	110.2(4)
P(2)-O(5)	1.497(7)	O(6)-P(3)-O(3)#1	108.5(4)
P(2)-O(1)	1.508(8)	O(11)-P(3)-O(3)#1	110.2(4)
P(2)-O(4)	1.515(7)	O(3)-P(3)-O(3)#1	110.8(6)
P(2)-O(9)	1.520(6)	O(3)-Mg/Al(1)-O(5)#2	109.6(4)
P(3)-O(6)	1.493(11)	O(3)-Mg/Al(1)-O(1)	110.0(3)
P(3)-O(11)	1.512(11)	O(5)#2-Mg/Al(1)-O(1)	108.2(3)
P(3)-O(3)	1.519(7)	O(3)-Mg/Al(1)-O(8)	107.8(4)
P(3)-O(3)#1	1.519(7)	O(5)#2-Mg/Al(1)-O(8)	111.2(4)
Mg/Al(1)-O(3)	1.842(7)	O(1)-Mg/Al(1)-O(8)	110.0(3)
Mg/Al(1)-O(5)#2	1.846(8)	O(10)-Al(2)-O(7)#3	111.3(4)
Mg/Al(1)-O(1)	1.852(8)	O(10)-Al(2)-O(2)#4	105.1(4)
Mg/Al(1)-O(8)	1.873(7)	O(7)#3-Al(2)-O(2)#4	110.5(4)
Al(2)-O(10)	1.737(8)	O(10)-Al(2)-O(9)	110.0(4)
Al(2)-O(7)#3	1.739(8)	O(7)#3-Al(2)-O(9)	110.4(4)
Al(2)-O(2)#4	1.743(8)	O(2)#4-Al(2)-O(9)	109.4(4)
Al(2)-O(9)	1.750(7)	O(6)#5-Al(3)-O(11)	110.0(4)
Al(3)-O(6)#5	1.752(11)	O(6)#5-Al(3)-O(4)#1	107.8(3)
Al(3)-O(11)	1.760(11)	O(11)-Al(3)-O(4)#1	111.8(3)
Al(3)-O(4)#1	1.761(7)	O(6)#5-Al(3)-O(4)	107.8(3)
Al(3)-O(4)	1.761(7)	O(11)-Al(3)-O(4)	111.8(3)
		O(4)#1-Al(3)-O(4)	107.5(6)
O(8)-P(1)-O(10)	111.0(5)	P(2)-O(1)-Mg/Al(1)	145.4(5)
O(8)-P(1)-O(7)	111.7(5)	P(1)-O(2)-Al(2)#6	147.2(5)
O(10)-P(1)-O(7)	108.2(4)	P(3)-O(3)-Mg/Al(1)	142.7(5)
O(8)-P(1)-O(2)	110.2(5)	P(2)-O(4)-Al(3)	143.2(5)
O(10)-P(1)-O(2)	105.6(4)	P(2)-O(5)-Mg/Al(1)#3	148.4(4)
O(7)-P(1)-O(2)	109.9(4)	P(3)-O(6)-Al(3)#7	177.6(7)
O(5)-P(2)-O(1)	109.2(4)	P(1)-O(7)-Al(2)#2	150.4(5)
O(5)-P(2)-O(4)	110.6(4)	P(1)-O(8)-Mg/Al(1)	140.1(5)
O(1)-P(2)-O(4)	109.9(4)	P(2)-O(9)-Al(2)	145.4(5)
O(5)-P(2)-O(9)	109.8(4)	P(1)-O(10)-Al(2)	151.7(4)
O(1)-P(2)-O(9)	111.9(4)	P(3)-O(11)-Al(3)	156.1(7)

Symmetry transformations used to generate equivalent atoms: #1 x,y,-z+3/2; #2 -x+y+2,-x,z; #3 -y,x-y-2,z; #4 y+1,-x+y+1,-z+1; #5 -x+y+2,-x+1,z; #6 x-y,x-1,-z+1; #7 -y+1,x-y-1,z.

Supplementary Table 6 | Crystal data and structure refinement details for JU-61.

Identification code	JU-61
Empirical formula	C Al1.50 O10.17 P2.50 Zn
Formula weight	357.94
Temperature	296(2) K
Wavelength	0.71073 Å
Crystal system	Trigonal
Space group	*R*3*m*
Unit cell dimensions	a = 13.448(3) Å α = 90°.
	b = 13.448(3) Å β = 90°.
	c = 38.562(8) Å γ = 120°.
Volume	6040(3) $Å^3$
Z	18
Density (calculated)	1.771 Mg/m^3
Absorption coefficient	2.256 mm^{-1}
F(000)	3138
Theta range for data collection	2.043 to 25.337°.
Index ranges	$-16 \leq h \leq 14, -13 \leq k \leq 16, -30 \leq l \leq 46$
Reflections collected	10427
Independent reflections	2500 (*R*(int) = 0.0505)
Completeness to theta = 25.242°	99.7 %
Refinement method	Full-matrix least-squares on F^2
Data / restraints / parameters	2500 / 7 / 163
Goodness-of-fit on F^2	1.430
Final *R* indices (I>2sigma(I)) *	*R*1 = 0.1128, *wR*2 = 0.3280
R indices (all data) *	*R*1 = 0.1321, *wR*2 = 0.3458
Largest diff. peak and hole	1.933 and −1.988 e.$Å^{-3}$

* $w = 1/(\sigma^2(Fo^2) + (0.2P)^2)$ where $P = (Fo^2 + 2Fc^2)/3$

Supplementary Table 7 | Atomic Coordinates (×10^4) and equivalent isotropic displacement parameters (Å^2 ×10^2) for JU-60. The "T" sites are co-occupied by tetrahedrally coordinated P, Al, and Zn.

Atom	*x*	*y*	*z*	*U*(eq)
T(1)	-8(4)	7607(4)	813(1)	16(1)
T(2)	1(6)	7671(5)	3(1)	33(1)
T(3)	3(4)	7616(3)	1985(1)	14(1)
T(4)	-959(5)	5724(4)	1396(2)	45(2)
T(5)	990(4)	6673(5)	-536(1)	27(1)
O(1)	250(14)	6820(17)	-257(5)	53(5)
O(2)	-310(30)	6430(20)	1103(6)	123(12)
O(3)	0(30)	7200(20)	380(7)	78(7)
O(4)	-1206(12)	7590(20)	-63(8)	52(8)
O(5)	1233(14)	8767(14)	939(8)	66(10)
O(6)	-2284(12)	5430(20)	1380(11)	88(14)
O(7)	-120(20)	6496(17)	1762(4)	58(5)
O(8)	1013(11)	8987(11)	-65(7)	49(7)
O(9)	1345(13)	8655(13)	1864(6)	74(10)
O(10)	820(20)	5412(11)	-432(6)	38(6)
O(11)	-897(16)	8210(30)	858(10)	82(11)
O(12)	-1010(20)	4496(10)	1418(6)	39(5)
O(13)	608(15)	6792(17)	-923(5)	46(4)
O(14)	-988(13)	8020(30)	1893(8)	65(9)
O(15)	2349(12)	7651(12)	-481(7)	52(8)
O(1W)	0	10000	1342(11)	60(12)
C(1)	-657(14)	8690(30)	-889(9)	34(7)
C(2)	631(15)	9369(15)	-1016(10)	42(8)

Supplementary Table 8 | Selected bond distances (Å) and angles (°) for JU-61. The "T" sites are co-occupied by tetrahedrally coordinated P, Al, and Zn.

T(1)-O(5)	1.688(11)	O(7)-T(3)-O(9)	100.6(11)
T(1)-O(11)	1.752(16)	O(7)-T(3)-O(14)	116.2(13)
T(1)-O(3)	1.76(3)	O(9)-T(3)-O(14)	110.9(14)
T(1)-O(2)	1.81(3)	O(7)-T(3)-O(13)#1	102.3(9)
T(2)-O(3)	1.58(2)	O(9)-T(3)-O(13)#1	115.3(11)
T(2)-O(4)	1.592(9)	O(14)-T(3)-O(13)#1	111.1(11)
T(2)-O(8)	1.627(10)	O(2)-T(4)-O(12)	114.3(15)
T(2)-O(1)	1.676(19)	O(2)-T(4)-O(6)	111(2)
T(3)-O(7)	1.670(18)	O(12)-T(4)-O(6)	106.0(11)
T(3)-O(9)	1.705(9)	O(2)-T(4)-O(7)	103.6(13)
T(3)-O(14)	1.713(11)	O(12)-T(4)-O(7)	104.0(11)
T(3)-O(13)#1	1.74(2)	O(6)-T(4)-O(7)	118.1(17)
T(4)-O(2)	1.46(3)	O(1)-T(5)-O(13)	111.9(11)
T(4)-O(12)	1.622(6)	O(1)-T(5)-O(10)	101.6(12)
T(4)-O(6)	1.623(9)	O(13)-T(5)-O(10)	116.2(11)
T(4)-O(7)	1.78(2)	O(1)-T(5)-O(15)	109.8(12)
T(5)-O(1)	1.54(2)	O(13)-T(5)-O(15)	108.7(12)
T(5)-O(13)	1.61(2)	O(10)-T(5)-O(15)	108.4(13)
T(5)-O(10)	1.645(8)	T(5)-O(1)-T(2)	150.0(13)
T(5)-O(15)	1.647(9)	T(4)-O(2)-T(1)	148(2)
		T(2)-O(3)-T(1)	138.8(14)
O(5)-T(1)-O(11)	97.7(15)	T(2)-O(4)-T(2)#2	160(2)
O(5)-T(1)-O(3)	113.8(15)	T(1)-O(5)-T(1)#3	146(2)
O(11)-T(1)-O(3)	110.0(15)	T(4)-O(6)-T(4)#2	155(2)
O(5)-T(1)-O(2)	108.4(14)	T(3)-O(7)-T(4)	132.9(13)
O(11)-T(1)-O(2)	115.9(16)	T(2)-O(8)-T(2)#3	148.4(19)
O(3)-T(1)-O(2)	110.6(9)	T(3)-O(9)-T(3)#3	139.8(16)
O(3)-T(2)-O(4)	108.5(16)	T(5)#4-O(10)-T(5)	148.8(16)
O(3)-T(2)-O(8)	114.6(15)	T(1)#2-O(11)-T(1)	132(2)
O(4)-T(2)-O(8)	108.6(13)	T(4)#4-O(12)-T(4)	172.7(18)
O(3)-T(2)-O(1)	104.1(13)	T(5)-O(13)-T(3)#5	138.9(12)
O(4)-T(2)-O(1)	113.6(13)	T(3)-O(14)-T(3)#2	139.5(18)
O(8)-T(2)-O(1)	107.6(12)	T(5)-O(15)-T(5)#3	145.2(19)

Symmetry transformations used to generate equivalent atoms: #1 -y+2/3,x-y+4/3,z+1/3; #2 -x+y-1,y,z; #3 -y+1,-x+1,z; #4 x,x-y+1,z; #5 -x+y-2/3,-x+2/3,z-1/3.

Supplementary Table 9 | Calculated reaction barriers and reaction energies for the methylation of hexamethylbenzene within eight ABC-6 cages.

Cage	$N_{8\text{-ring}}/N_{\text{layer}}$*	Reaction barrier (kcal mol^{-1})	Reaction energy (kcal mol^{-1})
(abbcca) / the chabazite cage	1.0	23.0	−0.4
(abbcbba)	0.86	19.9	0.9
(abbccba)	0.86	22.2	4.2
(abbcbbca)	0.75	20.0	−0.3
(abbcbcca)	0.75	21.4	6.2
(abbccbba)	1.125	19.9	−5.4
(abbccbca)	0.75	19.5	−5.7
(abccbbca)	0.75	26.7	−4.8

* $N_{8\text{-ring}}$ and N_{layer} are the number of 8-ring windows and the number stacking layers in an ABC-6 cage, respectively.

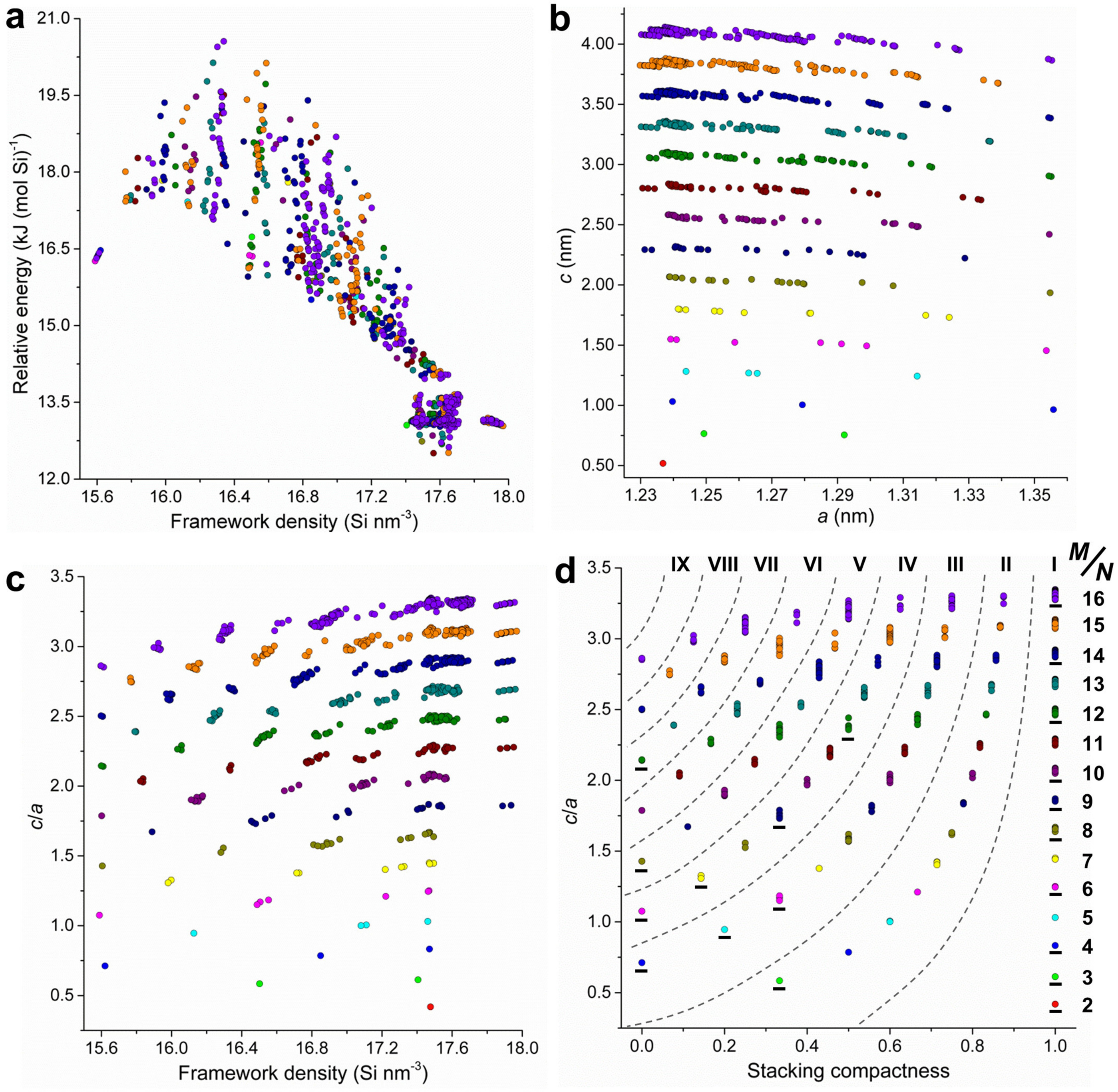

Supplementary Figure 1 | Relationships among framework energy, framework density, lattice dimensions, and stacking compactness for 1,150 ABC-6 models comprised of ≤4 types of constituent cages. a, framework energy versus framework density. **b**, cell dimensions *c* versus *a*. **c**, *c*/*a* versus framework density. **d**, *c*/*a* versus stacking compactness. Each individual group can be named according to the number of stacking layers (*N*) and the rank of its corresponding stacking compactness (*M*). Groups having at least one end member realized already are underlined with short bars. ABC-6 models comprised of different numbers of stacking layers are shown in different colours in these four plots.

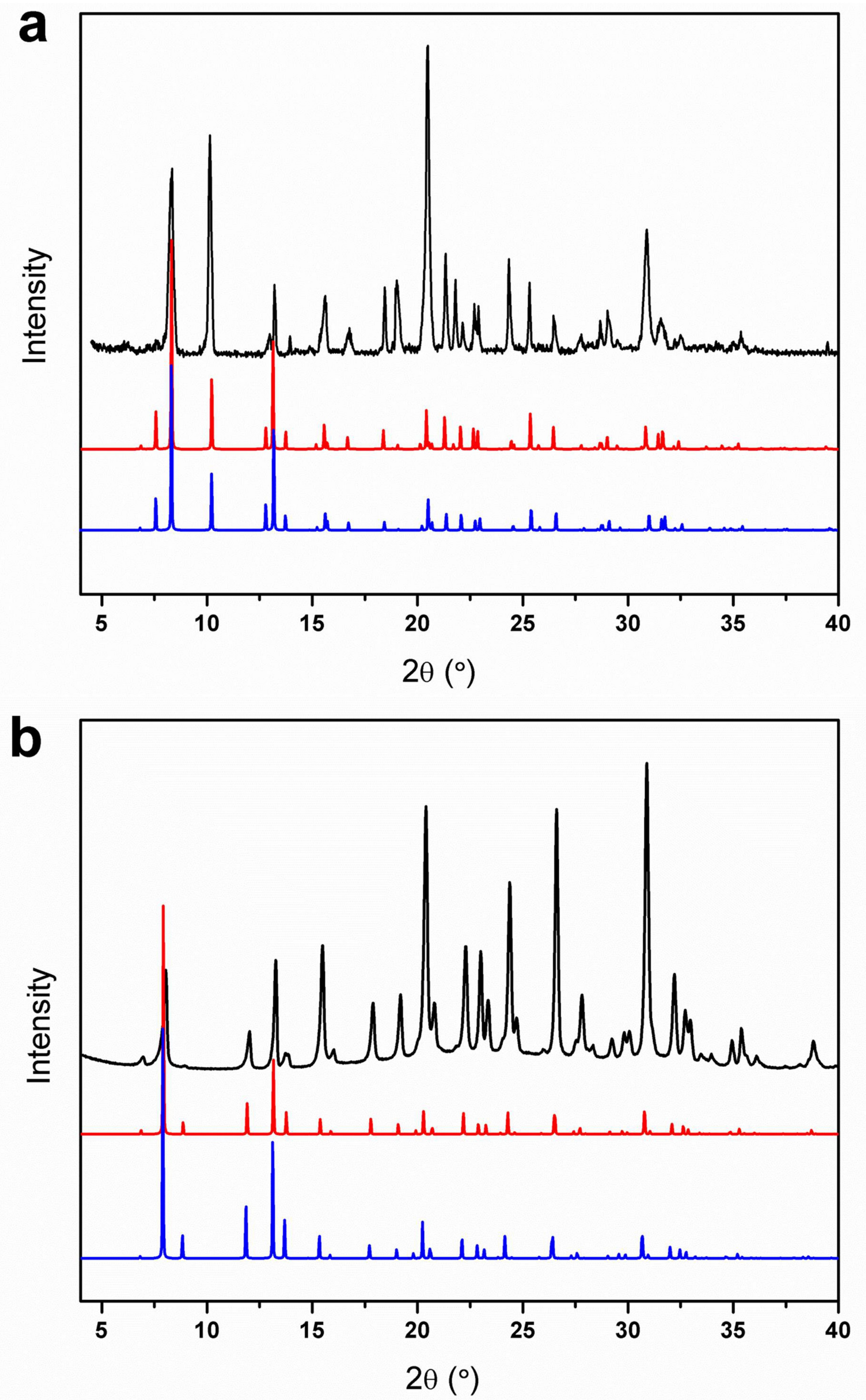

Supplementary Figure 2 | Experimental and simulated powder X-ray diffraction patterns for JU-60 (a) and JU-61 (b). The black patterns are experimental observations, the red patterns are simulated from single-crystal structures, and the blue patterns are simulated from the enumerated silica models that have been rescaled according to the ratios of Mg/Zn to Al.

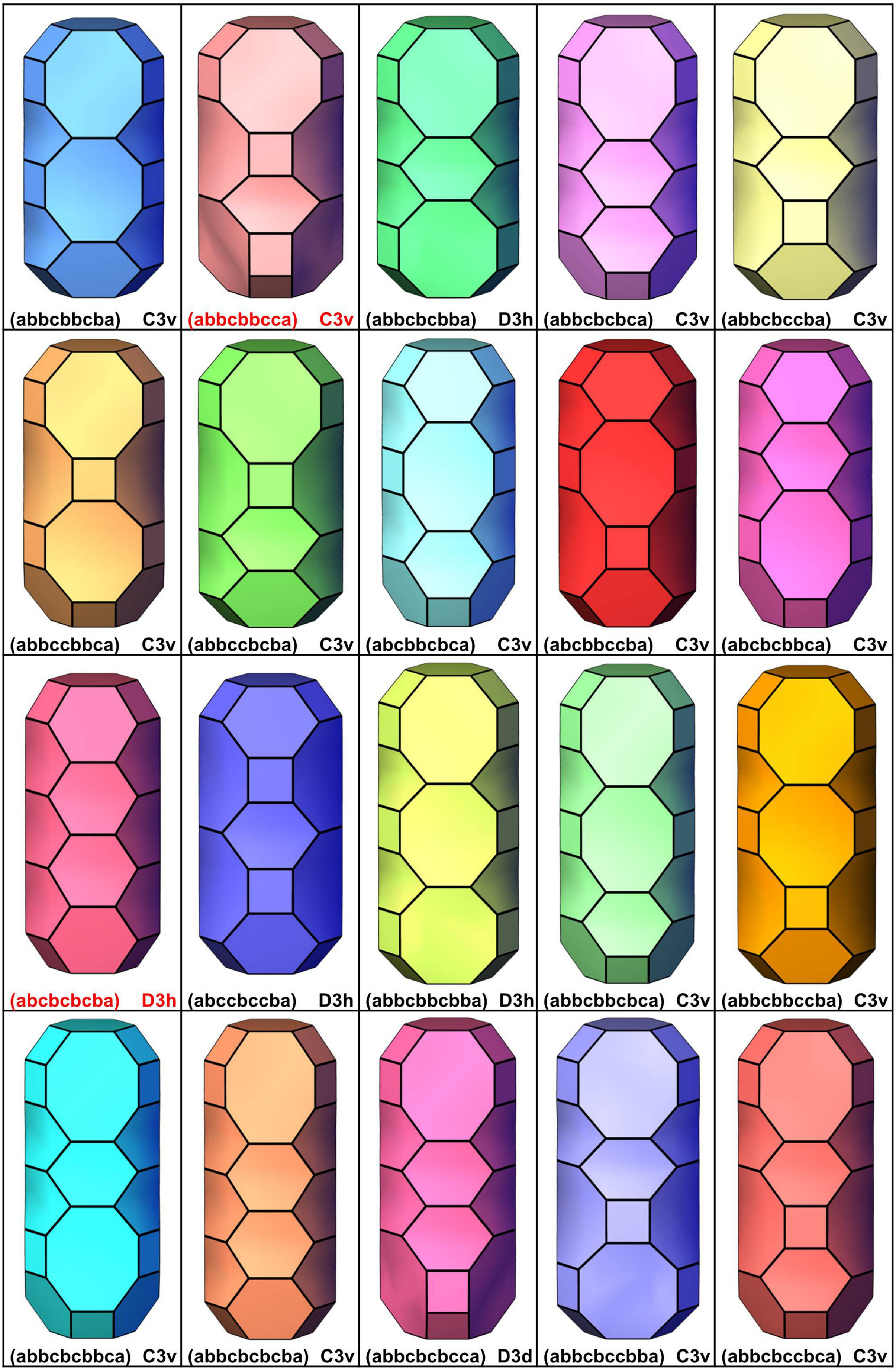

Supplementary Figure 3 | Enumerated ABC-6 cages constructed by 9 and 10 6-ring layers. For each cage, the stacking sequence and the highest allowed symmetry are given. The stacking sequences representing the cages that have already been observed in existing ABC-6 materials are highlighted in red.

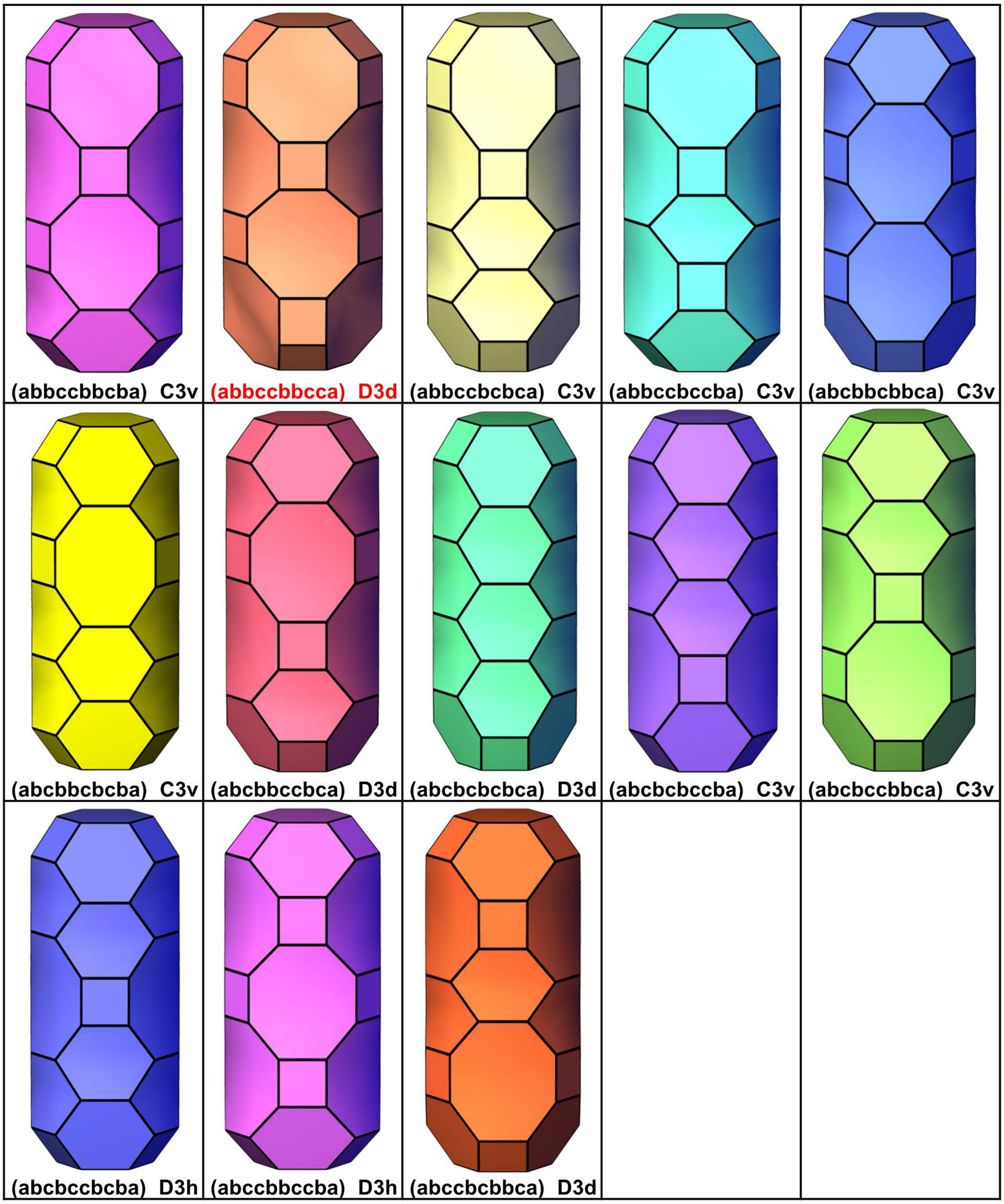

Supplementary Figure 3 (continued)

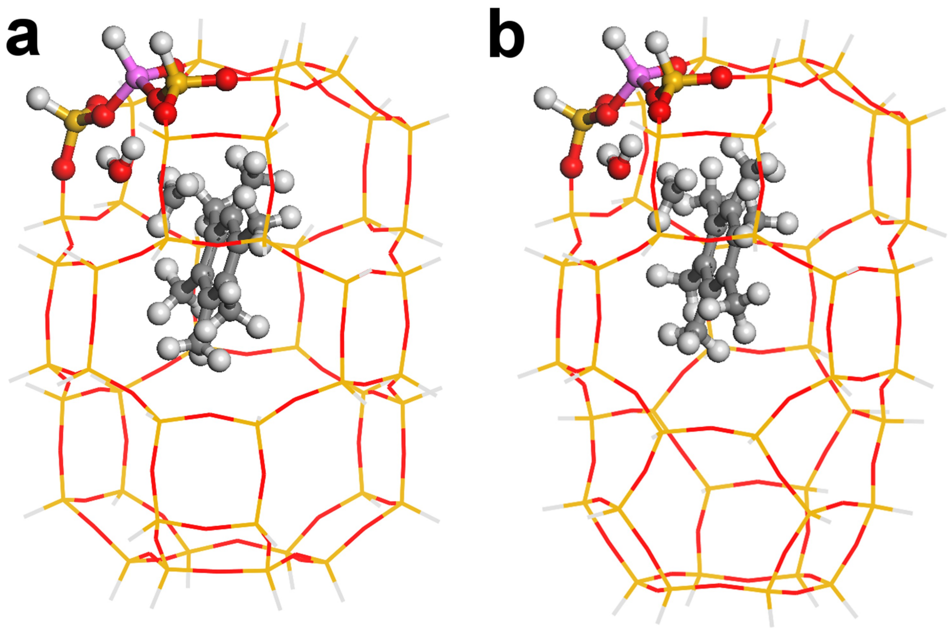

Supplementary Figure 4 | Transition states for the methylation of hexamethylbenzene within (a) the (abbccbba) cage and (b) the (abbccbca) cage. Ball-and-stick models show the high level part for ONIOM calculation. Colour code: yellow, Si; red, O; pink, Al; grey, C; white, H.
